# Water Temperature Effect on Flexural Strength of Posterior Ankle Splint: An Experimental Study

**DOI:** 10.5704/MOJ.2407.011

**Published:** 2024-07

**Authors:** C Pirot, N Sirimahatthanakul, A Naowanirut, T Sirithiantong

**Affiliations:** Department of Orthopaedic Surgery, Hatyai Hospital, Songkhla, Thailand

**Keywords:** splint, temperature, material, flexural strength, foot and ankle injuries

## Abstract

**Introduction:**

Plaster of Paris splints are commonly utilised for foot and ankle injuries. However, during follow-ups, some of these splints were found to be broken. Various methods, including splint form or augmentation changes, have been explored to enhance flexural strength. However, the impact of water temperature on the splint's flexural strength still needs to be studied. This research aimed to investigate the effect of water temperature on the flexural strength of the Plaster of Paris splint.

**Materials and Methods:**

Three groups were set up based on different water temperatures: cold, hot, and room temperature. Posterior ankle splints were created and immersed in water at these varying temperatures, with five pieces tested per group. The splints were then allowed to harden fully over three days. Each splint underwent a tensile strength test using an axial pressure machine, which recorded their flexural strength data.

**Results:**

There were no statistically significant differences in the general characteristics of the splints. The flexural strengths of the three splint groups (pre-cooled, pre-heated, and room temperature) were 182.6N, 162.45N, and 228.91N, respectively. Statistical analysis revealed that room-temperature splints demonstrated a statistically significant increase in flexural strength compared to pre-heated splints (p<0.05). However, they did not differ significantly from pre-cooled splints.

**Conclusion:**

The highest flexural strength was observed in splints immersed in room-temperature water.

## Introduction

Patients with foot and ankle injuries are frequently treated with posterior ankle splints made from Plaster of Paris (POP)^1^. However, some splints were found to be broken during follow-up appointments. These were common occurrences despite advising patients to avoid weight-bearing activities due to spontaneous dynamic contraction of the gastrocnemius muscle^2^. Broken splints not only incur unnecessary expenses but also lead to poor treatment outcomes. Flexural strength^3^, also known as modulus of rupture, bend strength, or transverse rupture strength, is a crucial material property. According to a previous study^4^, the force that significantly affects the failure of a posterior ankle splint is tensile strength. This study identified the inner side (tension side) at the heel corner of the splint as the starting point of initial failure.

However, posterior ankle splint tolerance to compression strength is better than tensile strength. Previous studies have focused on enhancing splint strength by changing its form^5^, increasing the layer of plaster of Paris^6^, or through augmentation^7,8^. Despite these efforts, there is a lack of research on the impact of water temperature on the flexural strength of splints.

From a review of relevant literature, a study of the preheated femoral stem in cemented total hip arthroplasty^9-11^ showed that the preheated stem causes the inner side of the cement mantle to be heater than the outer side. Consequently, the inner side of the cement mantle hardens before the outer side, resulting in a shift of porosity from the inner surface of the cement mantle to the outer side.

This technique showed stronger and reduced the rate of fatigue failure of the inner side (stem cement interface). Many laboratory studies have shown pores acting as fatigue crack initiation^12^. However, it was found that the high temperature of the water decreased the setting time of the splint^13^. The high-water temperature during splint immersion decreased the setting time and accelerated the hardening process, while low water temperature prolonged the setting time and slowed down the hardening process^13^.

The hypothesis in this study simulated the actual wearing of the posterior ankle splint in humans. If the temperature was increased on the inner side of the splint, the inner side of the splint began to harden before the outer side of the splint. This would cause the porosity of the splint to shift from the inner side of the splint to the outside of the splint, making the inner splint stronger and improving flexural strength.

We anticipate that the splint will exhibit increased strength. Consider the materials of bone cement, polymethyl methacrylate, and POP, which are gypsum or calcium sulfate^14^. They are two different materials. However, some properties are similar, such as being a solid material; there is also an exothermic reaction and porosity in the same material. The researcher expected that both materials could be applied to research.

We aimed to study the effect of water temperature on the flexural strength of the splint.

## Materials and Methods

**Fig. 1:**
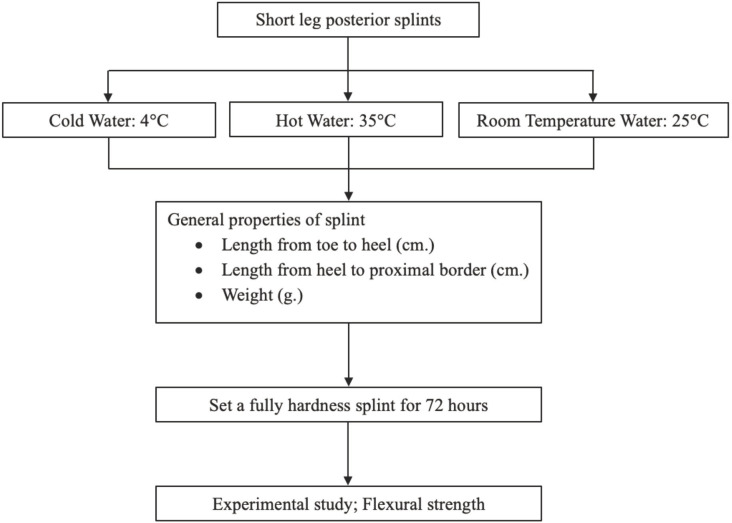
Study flow diagram.

This experimental protocol was approved by the institutional ethics committee (Protocol no. HYH EC 011-66-01) and was conducted in accordance with the Declaration of Helsinki. The study flow is depicted in Fig. 1.

We examined three groups of splints, each containing five splints immersed in water at different temperatures. This resulted in a total of 15 POP splints. These 15 splints were prepared using 10-layer, 4-inch-wide POP materials [POP LOT no. 228, manufactured in July 2022 by BSN MEDICAL SAS®, France]^15^. The splints were 17 inches long, corresponding to the size of the leg mannequin used in this study.

**Fig. 2:**
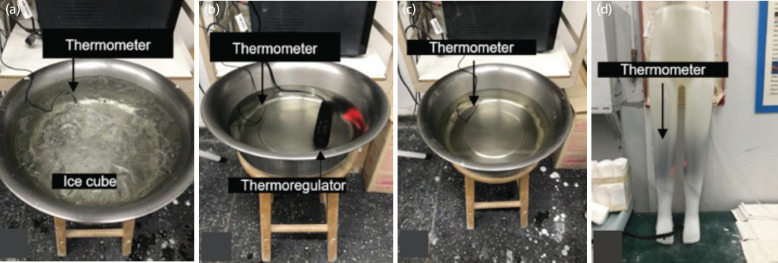
(a) Cold water, (b) hot water, (c) room-temperature water, and (d) leg mannequin.

The splints were immersed in three different water groups: precooled splint (Fig. 2a) at a temperature of 4°C (this water temperature can be achieved by placing ice cubes into a water bath. It can make the water temperature 4°C. At 4°C, water is still in its liquid form^16^. several tests in this study have been conducted to measure the temperature of ice cubes in a water bath shown as 4°C) and monitor the temperature with a thermometer [RC-4, Elitech®, London, UK]; preheated splint (Fig. 2b) set to 35°C (thermoregulator can be set the maximum temperature at 35°C. Furthermore, safe temperature applies to humans (more than 40°C produces skin burn^17,18^), maintained using a thermoregulator [GR-100B, Sunsun®, Zhejiang, China], with temperature verified using a thermometer, and room temperature splint (Fig. 2c) set as temperature of 25°C [The International Union of Pure and Applied Chemistry (IUPAC) defines standard ambient temperature as 25°C]^19^, again monitored with a thermometer. A leg mannequin (Fig. 2d) replicates a patient's leg. This hollow-leg mannequin was filled with water, and a thermoregulator was used to maintain an internal temperature of 35°C. The surface temperature of the mannequin, measured by a thermometer, was 33°C, which is similar to the average human skin temperature^20^.

Fresh water was used for each splint to prevent contamination from residual plaster. After immersion, each splint was drained and moulded to the prepared leg mannequin. The splint was placed on the leg mannequin for 5 minutes before removal. Subsequently, all splints were stored at room temperature for 72 hours to ensure complete hardening ^21^.

The length and weight of each splint were measured next. There were two length measurements for each splint: from the toe to the heel and from the heel to the upper border of the splint. A tape measure was used to record these measurements, with two readings taken for each and their average calculated. The weight of each splint was determined using a digital scale.

The strength of the splints was tested using a tensile testing machine [Z005, Zwick Roell®, Germany]. The splints were subjected to axial pressure at a rate of 10mm per minute, and the force necessary to bend each splint was recorded. Testing is done by increasing axial compression on the specimen and watching until the amount of axial compression exerted gradually increases to the maximum axial compression until the workpiece begins to bend at the heel position. Subsequently, the applied axial compression was reduced. The maximum axial compression is defined as the flexural strength of the posterior ankle splint^22^.

Data were analysed using Stata version 15.1 [StataCorp LLC, College Station, TX, USA]. Mean values, standard deviations, and 95% confidence intervals were reported for the data sets. Comparisons of length, splint weight, and the force required to bend a splint were conducted using an ANOVA test, with a statistical significance threshold set at p<0.05.

## Results

Based on the data collected, there were no statistically significant differences in the general properties of the splints among the three experimental groups. This included variables such as the length from toe to heel, the length from heel to upper border, and the weight of the splints (p>0.05). However, when examining the flexural strength across all three groups using the ANOVA method, a significant effect of water temperature on the flexural strength of the splints was observed (P=0.028). Detailed results are presented in Table I.

**Table I:** General characteristics and experimental conditions of the tested splints.

General characteristic data	Pre-cooled splint	Pre-heated splint	Room-temperature splint	P-value
Length from toe to heel (cm) (mean ± SD)	12.5 ± 0.2	12.4 ± 0.2	12.3 ± 0.2	0.147
Length from heel to upper border (cm) (mean ± SD)	32.2 ± 0.2	32.2 ± 0.2	32.4 ± 0.3	0.653
Weight (g) (mean ± SD)	475.8 ± 4.1	475.2 4.8	3.8	0.953
Flexural strength (N) (mean ± SD)	182.6 ± 29.8	162.5 25.2	228.9 ± 45.5	0.028

The analysis of the flexural strength (N) of the splints across the three experimental groups, each with different water temperatures, was analysed using ANOVA with Bartlett's test for equal variances. The variances were found to be equal across all groups. Post-hoc statistical analysis revealed that splints dipped in room-temperature water (room temperature splint) exhibited significantly greater flexural strength compared to those dipped in hot water (preheated splint) (mean difference 66.4, p=0.031). However, there was no statistically significant difference in flexural strength between splints dipped in room- temperature water (room temperature splint) and those dipped in cold water (precooled splint). Detailed results are displayed in Table II.

**Table II:** Post-Hoc test; comparison of flexural strength by test group (Bonferroni).

Compared between group	(Mean difference, p-value)	
	Pre-cooled splint	Room-temperature splint
Room temperature splint	46.3114, 0.168	
Pre-heated splint	-20.1479, 1.000	-66.4593, 0.031

## Discussion

The effects of temperature on POP setting time are well-recognised^13^; however, the effects of water temperature on splint strength were unknown. Our results indicate that temperature influences the flexural strength of the posterior ankle splint. In this study, splints were immersed in water at different temperatures: 4, 25, and 35°C, while the inner layer of the splint came into contact with a temperature of 33°C (similar to the average human skin temperature). The temperature of the inner side surface difference between the temperature of 33°C of the leg mannequin and splints immersed in water at different temperatures. This is explained as follows: (1) The splints were immersed in cold water (the difference temperature of the inner side of the splint increased to 31°C). The inner side of the splint temperature is more than the outer side of the splint. The inner side should harden before the outer side and shift porosity to the outer side. As a result, the inner side should have higher flexural strength than the outer side. (2) splints immersed in room temperature water (the difference temperature inner side of splint increases 10°C), the Inner side of the splint temperature more than the outer side of the splint. The inner side should harden before the outer side and shift porosity to the outer side. As a result, the inner side should have higher flexural strength than the outer side. (3) Splints dipped in hot water, (the difference temperature inside of the splint decreases by 2°C), experienced a lower inner side temperature, leading to delayed hardening and porosity shift to the inner side, resulting in lower flexural strength.

A posterior ankle splint that is dipped in room-temperature water demonstrated the highest flexural strength. Comparatively, a room-temperature splint had a significantly higher flexural strength than a pre-heated splint, while no significant difference was observed compared to a pre-cooled splint.

Several authors have examined the effect of temperature on the setting time of splints, noting a decrease in setting time with higher water temperatures, few have commented on the strength of the splints^17,18^. Our study is among the first to establish a correlation between water temperature and flexural strength. This finding could help prevent splint breakage, thereby improving treatment outcomes and reducing costs.

In the future, applying these findings to patients with foot or ankle injuries could prove beneficial. Ankle movements are often restricted after applying a posterior ankle splint, and involuntary contractions of the gastrosoleus muscle complex can lead to splint breakage.

Usually, the force of a muscle depends on its length. When the gastrocnemius muscle is positioned neutrally (as with a splint), the gastrocnemius tendon force is 731±57N. in the medial gastrocnemius and 306±24N. in the lateral gastrocnemius^23^. Dedicated muscle activation is less than 10% of maximal voluntary contraction^24^ (MVC). The total force of the MVC of the gastrocnemius in the neutral ankle position is 1077N. An involuntary or redundant muscle contraction will exert <107 N of force. This experimental result shows that splints prepared in different water temperatures can withstand flexural forces more significantly than 107N. However, it's important to note the limitations of this study, particularly the static nature of muscle force application in the testing machine compared to dynamic contractions in real-life situations.

This difference represents a study limitation. The tensile testing machine applies a continuous compression force that does not precisely mirror real-world, dynamic contractions and is unable to show porosity shift due to a lack of measurement instruments. Therefore, the research team suggests conducting fatigue tests and porosity shift measurements of splints in future studies.

## Conclusion

The inner side (tension side) at the heel corner of the posterior ankle splint was found to be the initial failure side. If the temperature was increased on the inner side of the splint, the inner side of the splint began to harden before the outer side of the splint. This would cause the porosity of the splint to shift from the inner side to the outside of the splint; this will make the inner splint stronger and improve flexural strength. We recommend dipping splints in room-temperature water to achieve optimal flexural strength and easy to apply in clinical situations. Care should be taken when using hot water for splint preparation as it may lead to decreased flexural strength.

## References

[1] Court-Brown CM, Heckman JD, McQueen MM, Ricci WM, Tornetta P III, McKee MD (2014). Rockwood and Green's Fractures in Adults.

[2] Nordin M, Frankel VH (2001). Basic biomechanics of the musculoskeletal system.

[3] Ashby MF (2004). Materials selection in mechanical design.

[4] Schmidt VE, Somerset JH, Porter RE (1973). Mechanical properties of orthopedic plaster bandages. J Biomech..

[5] Gluck MJ, Beck CM, Sochol KM, London DA, Hausman MR (2019). Comparative strength of elbow splint designs: a new splint design as a stronger alternative to posterior splints. J Shoulder Elbow Surg..

[6] Vieira GC, Barbosa RI, Marcolino AM, Shimano AC, Elui VM, Fonseca MC (2011). Influence of the number of layers of paris bandage plasters on the mechanical properties speciments used on orthopedic splints. Rev Bras Fisioter..

[7] Thapanakulsak D (2011). Effects of Various Modified Heel Slab Techniques on the Strength of Short Leg Posteri. Vajira Med J..

[8] Thompson SF, McBride C, Conant SH, Moore MC, Lewis TR (2020). Effect of Side Struts on the Strength of Long Arm Plaster Splints: A Biomechanical Study. J Pediatr Orthop..

[9] Wilairatana V, Pirot C, Limpaphayom N (2015). Effects of Cemented Hip Stem Pre-heating on Stem Push-out Strength. Orthop Surg..

[10] Iesaka K, Jaffe WL, Kummer FJ (2003). Effects of preheating of hip prostheses on the stem-cement interface. J Bone Joint Surg Am..

[11] Bishop NE, Ferguson S, Tepic S (1996). Porosity reduction in bone cement at the cement-stem interface. J Bone Joint Surg Br..

[12] Hoey D, Taylor D (2009). Quantitative analysis of the effect of porosity on the fatigue strength of bone cement. Acta Biomater..

[13] Boyd AS, Benjamin HJ, Asplund C (2009). Principles of casting and splinting. Am Fam Physician..

[14] DeMaio M, McHale K, Lenhart M, Garland J, McIlvaine C, Rhode M (2012). Plaster: our orthopaedic heritage: AAOS exhibit selection. J Bone Joint Surg Am..

[15] Halanski M, Nemeth BA, Noonan KJ (2014). Cast and splint immobilization, remodeling, and special issues of Children's fractures. Rockwood and Wilkins' Fractures in Children: Eighth Edition.

[16] Rafferty JP Why Does Water Freeze from the Top Down?. Britannica.

[17] Conroy SM, Ward D, Fraser J (2007). Laboratory investigation into optimal temperature for plaster of Paris application. Emerg Med Australas..

[18] Lavalette R, Pope MH, Dickstein H (1982). Setting temperatures of plaster casts. The influence of technical variables. J Bone Joint Surg Am..

[19] Helmenstine A (2020). What Is Room Temperature?. Science Notes and Projects.

[20] Lee CM, Jin SP, Doh EJ, Lee DH, Chung JH (2019). Regional Variation of Human Skin Surface Temperature. Ann Dermatol.

[21] Szostakowski B, Smitham P, Khan WS (2017). Plaster of Paris-Short History of Casting and Injured Limb Immobilzation. Open Orthop J..

[22] Tensile Testing An Introduction. Instron.

[23] Maganaris CN (2003). Force-length characteristics of the in vivo human gastrocnemius muscle. Clin Anat..

[24] Kishibuchi K, Kouzaki M (2013). Medial gastrocnemius is a key muscle for involuntary alternate muscle activity of plantar flexor synergists. Neurosci Lett..

